# Supplementary material for: Primary Colorectal Tumor Displays Differential Genomic Expression Profiles Associated with Hepatic and Peritoneal Metastases
Source: Cancers (Basel). 2023 Sep 4;15(17):4418. doi: 10.3390/cancers15174418 (PMC10648258; doi:10.3390/cancers15174418)
Supplement: Supplementary file 1 [file cancers-15-04418-s001.zip › cancers-2534275-supplementary.pdf]

Supplementary Figures and Tables

Supplementary Figure S1. Disease free survival (right-sided) and Overall (left-sided) survivals curves according to expression of ACE2 (a), CLDN18 (b) and DUSP4 (c) protein in primary tumor samples.

Supplementary Figure S2. Representative gene sets enriched in CRC-Peritoneum (a) and CRC-Liver (b) groups

Supplementary Figure S3. Pathological and genomic data associate to the CRC-Peritoneum signature in TCGA Firehose cohort: (a) Lymphovascular and vascular invasion rates (Chi square test); (b) OncoTree tumor classification system (Chi square test)

Supplementary Table S1. Differential expressed genes found between CRC with LM and CRC with PM identified by LIMMA algorithm

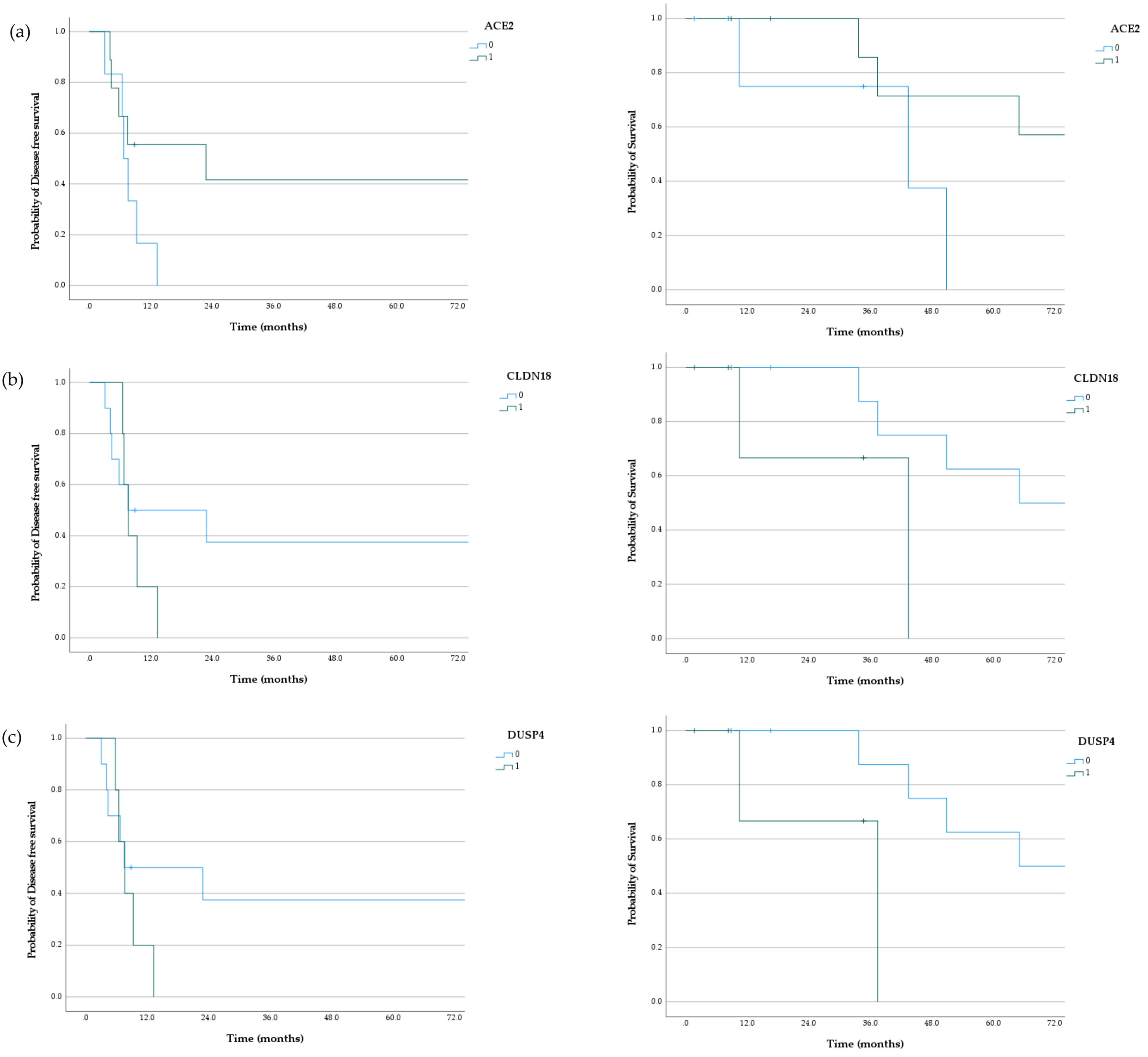

Supplementary Figure S1. Disease free survival (right-sided) and Overall (left-sided) survivals curves according to expression of ACE2 (a), CLDN18 (b) and DUSP4 (c) protein in primary tumor samples.

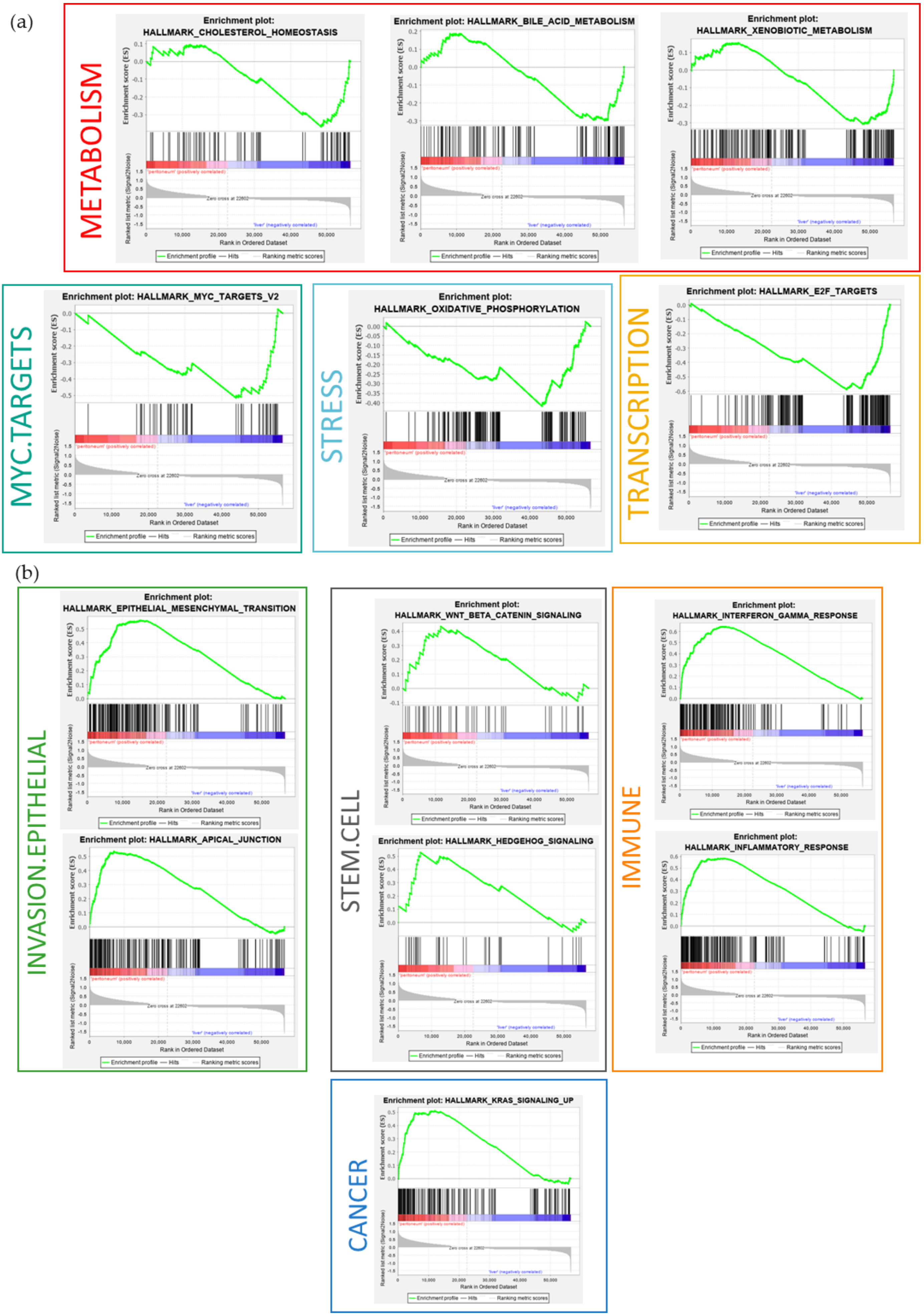

Supplementary Figure S2. Representative gene sets enriched in CRC-Liver (a) and CRC-Peritoneum (b) groups.

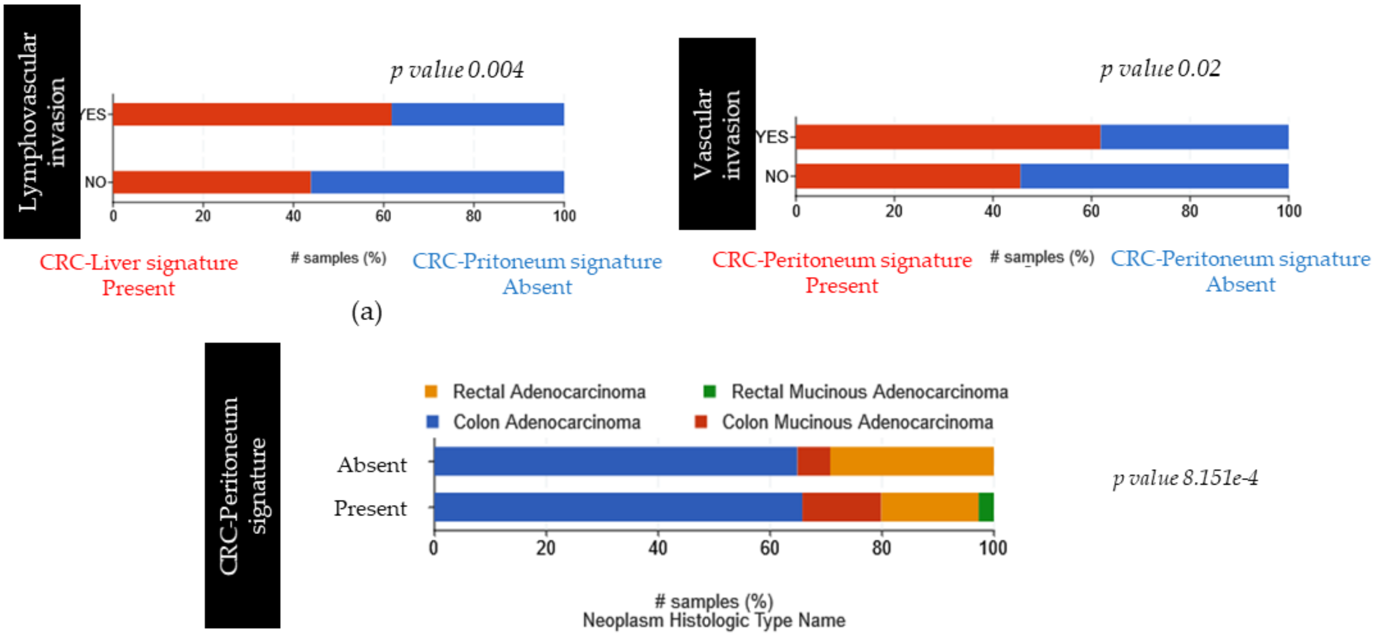

Supplementary Figure S3. Pathological and genomic data associate to the CRC-Peritoneum signature in TCGA Firehose cohort: (a) Lymphovascular and vascular invasion rates (Chi square test); (b) OncoTree tumor classification system (Chi square test)

Supplementary Table S1. Differential expressed genes found between CRC with LM and CRC with PM identified by LIMMA algorithm: respective significant genes are presented by their up regulation by class of metastasis, for each respective genes LIMMA output statistics are presented in different columns: logFC : logarithm 2 of fold change, AveExp : average expression, t statistics, raw p-values, False Discovery Rate adjusted p-values, and Bayes B statistics

Genes	logFC	AveExpr	t	P.Value	adj.P.Val	B
PLA2G12B	-5,615	-0,65	-6,716	0,00000	0,002770	6,603
CLDN18	4,891	-1,424	6,534	0,00000	0,002770	6,185
AC034229.2	4,57	-5,115	6,354	0,00000	0,002770	5,77
PKLR	-4,671	-1,087	-6,31	0,00000	0,002770	5,666
NTSR1	5,28	-1,476	6,193	0,00000	0,003060	5,395
CYP4F2	-4,003	1,195	-5,528	0,00001	0,016400	3,813
PRSS2	5,756	0,743	5,444	0,00001	0,016600	3,609
SLC26A9	4,647	-1,826	5,421	0,00001	0,016600	3,554
LINC02441	-4,795	-0,288	-5,309	0,00001	0,019300	3,283
TBR1	4,208	-3,69	5,289	0,00001	0,019300	3,235
AC097478.2	3,278	-5,027	5,193	0,00001	0,022900	3,003
KIF19	3,999	0,605	5,131	0,00002	0,023400	2,851
SLC13A2	-4,595	1,266	-5,127	0,00002	0,023400	2,842
TCN1	4,907	2,635	5,054	0,00002	0,026600	2,666
MSMB	4,308	-3,363	4,955	0,00003	0,032900	2,424
LINC01413	3,814	-5,235	4,899	0,00003	0,034100	2,289
ACE2	-3,267	4,819	-4,895	0,00003	0,034100	2,278
LDLRAD1	3,64	-1,811	4,869	0,00003	0,034100	2,216
C8orf37-AS1	2,765	-5,127	4,844	0,00004	0,034100	2,154
CELP	-3,05	1,817	-4,84	0,00004	0,034100	2,144
HRASLS5	3,754	-1,146	4,693	0,00006	0,049100	1,787
AC034229.3	-3,344	-4,197	-4,637	0,00007	0,054800	1,652
ROPN1L	2,712	-2,813	4,616	0,00007	0,055600	1,601
PLXNB3	3,68	1,785	4,586	0,00008	0,057900	1,526
GRIP2	2,796	1,202	4,563	0,00008	0,057900	1,473
ARMC4	2,986	-1,629	4,551	0,00008	0,057900	1,442
DUSP4	2,764	4,099	4,544	0,00009	0,057900	1,426
AC106772.2	-2,994	-4,959	-4,524	0,00009	0,059200	1,376
TNMD	-3,205	-1,405	-4,507	0,00009	0,059900	1,336
HAVCR1	-3,769	0,413	-4,409	0,00012	0,076100	1,099
HS3ST5	3,718	-2,673	4,366	0,00014	0,080700	0,994
C16orf54	3,036	1,156	4,365	0,00014	0,080700	0,991
KLK11	3,457	1,548	4,341	0,00015	0,081900	0,934
DDIT4L	3,401	-0,774	4,32	0,00016	0,081900	0,884
UGT2B17	-7,3	1,571	-4,31	0,00016	0,081900	0,859
ARX	3,749	-3,318	4,309	0,00016	0,081900	0,857
ULK4P1	3,357	-3,238	4,307	0,00016	0,081900	0,853

AQP12A	-3,424	-3,081	-4,286	0,00017	0,083800	0,803
CAPN13	-3,278	2,165	-4,28	0,00018	0,083800	0,787
FABP1	-4,724	7,552	-4,261	0,00019	0,083900	0,742
CCDC181	3,303	-3,274	4,252	0,00019	0,083900	0,721
AC004947.1	3,817	-3,246	4,244	0,00020	0,083900	0,7
LINC01589	2,706	-4,566	4,238	0,00020	0,083900	0,686
SCG2	3,005	2,233	4,236	0,00020	0,083900	0,681
FZD9	2,874	-1,365	4,224	0,00021	0,083900	0,653
HAND1	4,081	-3,397	4,216	0,00021	0,083900	0,634
LINC02084	2,879	-3,231	4,212	0,00021	0,083900	0,625
NBPF5P	3,251	-4,294	4,187	0,00023	0,086400	0,564
CYP2B6	-3,619	3,455	-4,186	0,00023	0,086400	0,562
AC019257.2	-2,658	-3,617	-4,161	0,00025	0,089600	0,502
CECR3	-3,074	-4,065	-4,158	0,00025	0,089600	0,494
AC004233.1	-2,774	-4,576	-4,14	0,00026	0,089600	0,452
AC093585.1	-3,006	-4,035	-4,138	0,00026	0,089600	0,447
PDE10A	2,725	2,295	4,133	0,00027	0,089600	0,434
BMPR1B	3,186	-0,21	4,132	0,00027	0,089600	0,431
TTC29	2,769	-4,038	4,12	0,00028	0,091000	0,402
AC097478.3	2,917	-5,534	4,093	0,00030	0,095200	0,337
AC007614.3	-3,007	-2,851	-4,084	0,00031	0,095200	0,318
CLEC18B	2,85	-1,124	4,084	0,00031	0,095200	0,316
PRKCG	3,19	-0,338	4,078	0,00031	0,095200	0,303
CTSG	3,181	-0,738	4,058	0,00033	0,098900	0,255
